# Chemical and Physical Properties of YHg_3_ and LuHg_3_

**DOI:** 10.1021/acsorginorgau.2c00048

**Published:** 2023-02-02

**Authors:** Kristian Witthaut, Yurii Prots, Nazar Zaremba, Mitja Krnel, Andreas Leithe-Jasper, Yuri Grin, Eteri Svanidze

**Affiliations:** Max-Planck-Institut für Chemische Physik fester Stoffe, Nöthnitzer Str. 40, 01187 Dresden, Germany

**Keywords:** amalgams, superconductivity, disorder, mercury, rare-earths, lanthanides

## Abstract

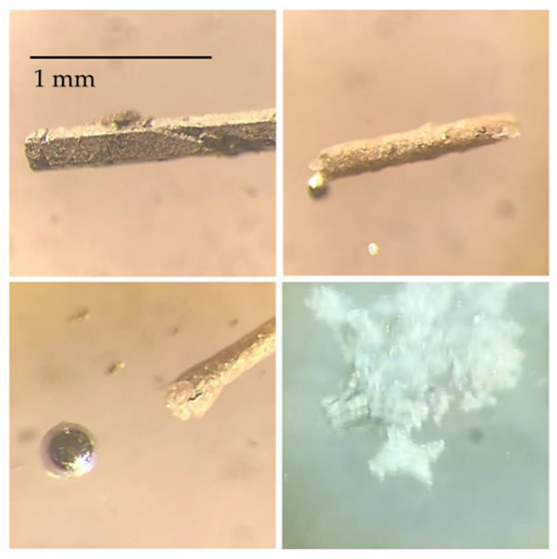

Amalgams have played an important role in fundamental and applied solid-state chemistry and physics because of the diversity of crystallographic features and properties that they have to offer. Moreover, their peculiar chemical properties can sometimes give rise to unconventional superconducting or magnetic ground states. In the current work, we present an in-depth analysis of single crystals of YHg_3_ and LuHg_3_ (Mg_3_Cd structure type, space group *P*6_3_/*mmc*). Both compounds show superconductivity below *T*_c_ = 1 ± 0.1 K (YHg_3_) and *T*_c_ = 1.2 ± 0.1 K (LuHg_3_). Given the high air-sensitivity and toxicity of these compounds, this study was only possible using a number of dedicated experimental techniques.

Among rare-earth elements, lutetium is the least soluble one in mercury.^1^ The binary phase diagram of Lu–Hg has not been investigated in detail.^2^ It has been suggested that the Lu–Hg system would likely look similar to that of Er–Hg and Ho–Hg.^2^ The Y–Hg phase diagram remains partially unknown.^3−7^ Among the Y–Hg and Lu–Hg phases, three compounds have been reported to exist: Y/LuHg (structure type CsCl),^5,8,9^ Y/LuHg_2_ (structure type UHg_2_),^6,9,10^ and Y/LuHg_3_ (structure type Mg_3_Cd).^4,9,10^ The latter structure type, with the *P*6_3_*/mmc* space group,^4,8−24^ is the most common among all compounds with the 1:3 and 3:1 stoichiometry,^18−22,25−29,30−35^ as summarized in Figure 1. The atomic positions are reversed in the latter case, which results in Na_3_Hg, Mg_3_Hg, and Cu_3_Hg compounds (elements marked with stars).^22,24,35^

**Figure 1:**
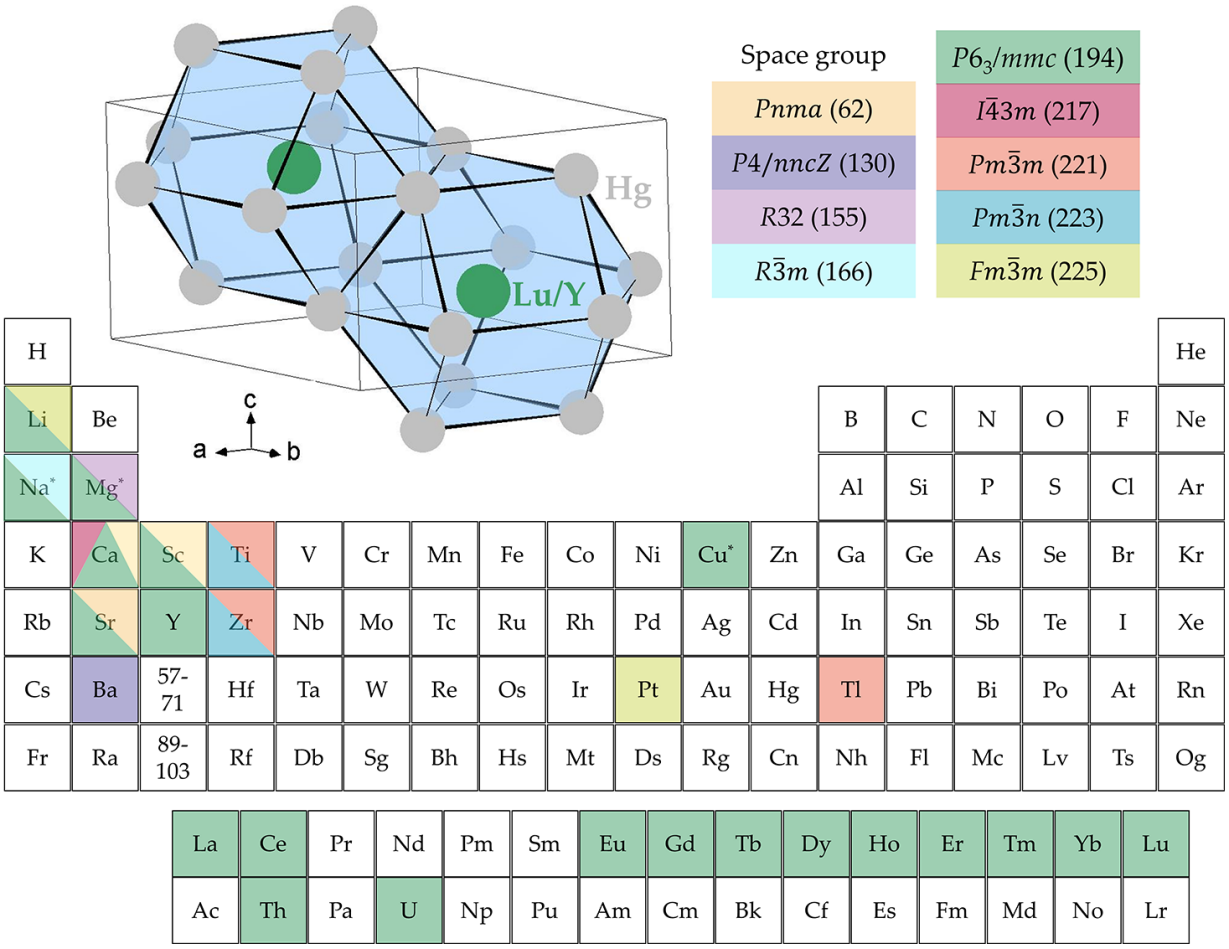
Space groups of binary mercury-based compounds with 1:3 and 3:1 (elements marked by stars) stoichiometries. Majority of compounds crystallize with the *P*6_3_/*mmc* space group.^4,8−34^ The inset shows the Mg_3_Cd-type crystal structure of Y/LuHg_3_ (*P*6_3_/*mmc* space group) with gray and green spheres representing Hg and Y/Lu, respectively.

Motivated by the lack of chemical and physical characterization of YHg_3_ and LuHg_3_, we performed a detailed study of these materials. It was possible to obtain large, millimeter-sized single crystals of YHg_3_ and LuHg_3_, both of which have needlelike morphology, see Figures 2 and 3. Unlike the Cu_3_Hg phase,^24^ both YHg_3_ and LuHg_3_ are highly air- and moisture-sensitive, decomposing quickly even after minute exposure to air. This is also the case for many other Hg-containing materials,^15,16,36−42^ which thus require a special synthesis and handling laboratory environment.^43^

**Figure 2:**
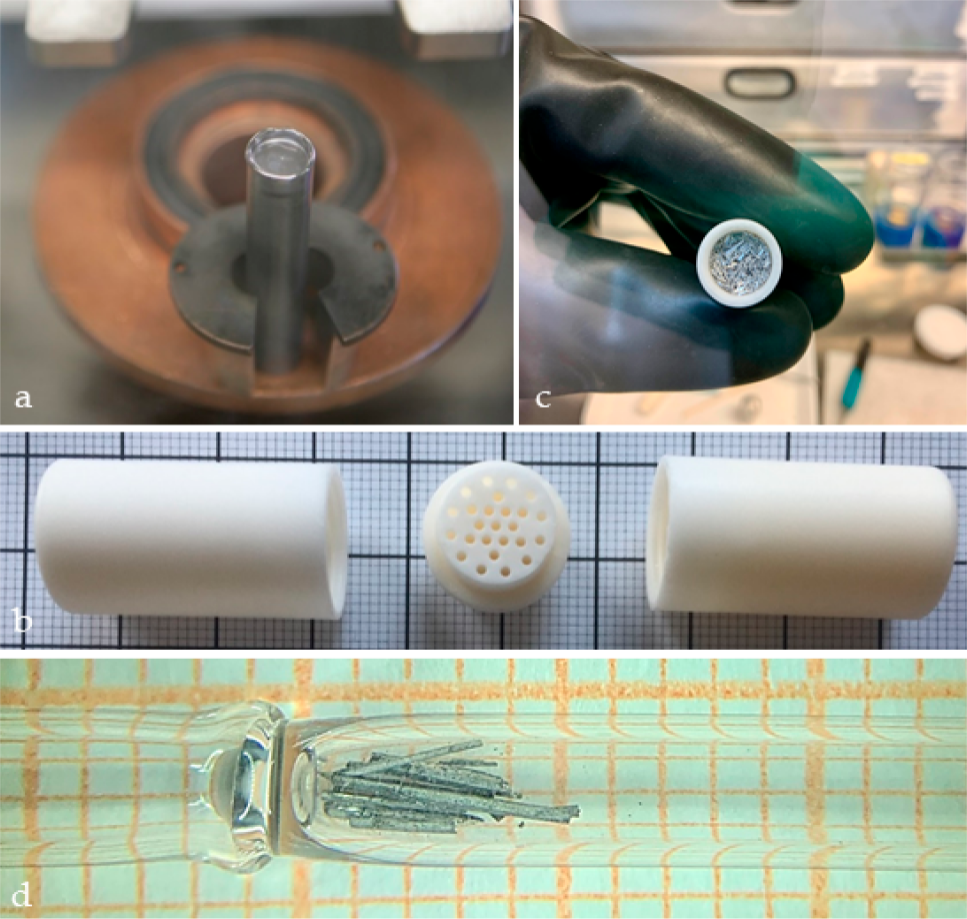
Synthesis of YHg_3_ and LuHg_3_ is carried out in a sealed Ta tube, with the excess Hg removed via centrifugation at room temperature (a). This is done using a custom-made alumina crucible, shown in (b–d). The resultant crystals have needlelike morphology with lengths of up to 1 cm.

**Figure 3:**
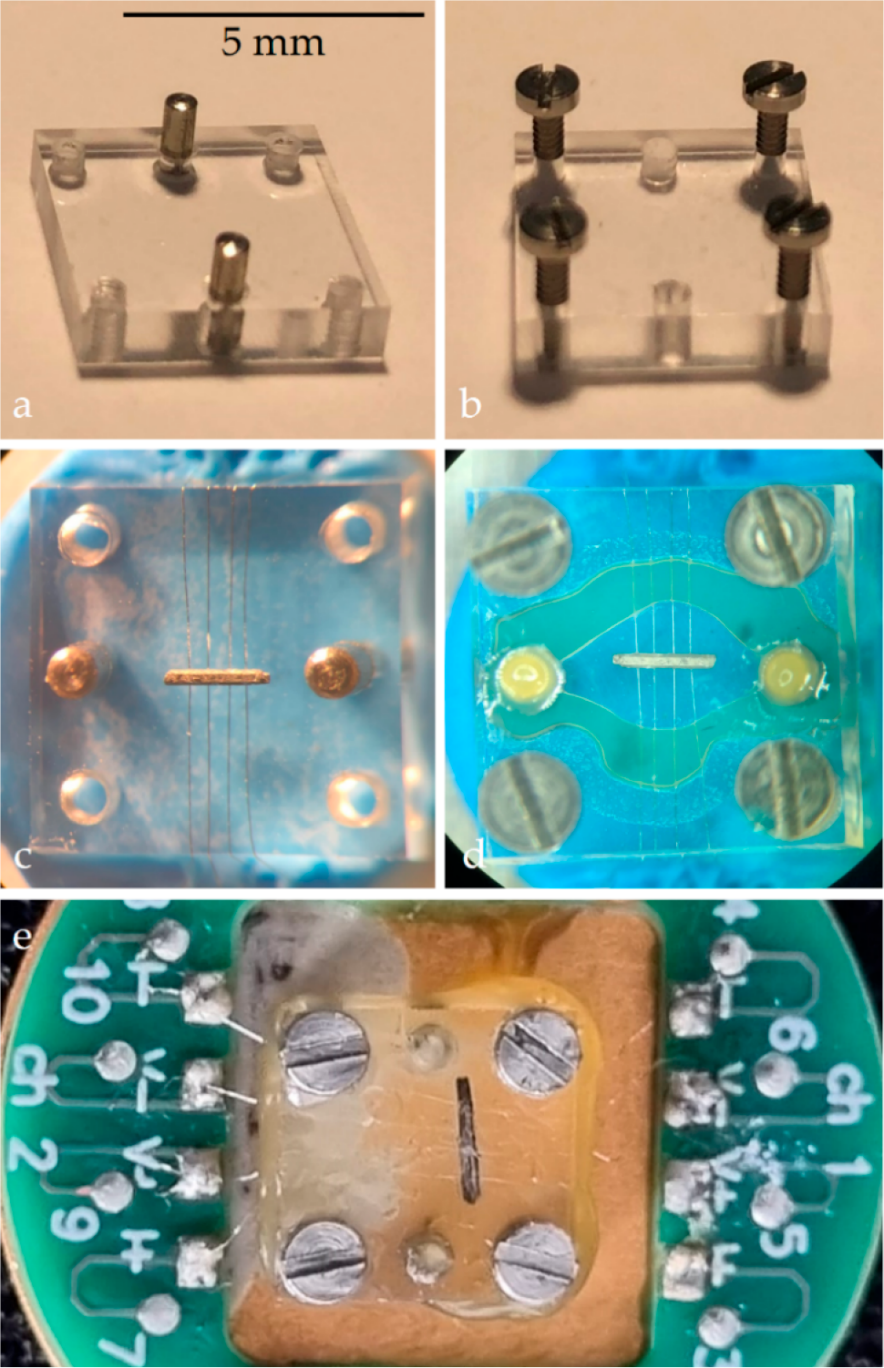
(a,b) The custom-made resistivity cell is composed of two platforms. The wires are placed on the bottom platform, and the sample is mounted on top of the wires, as shown in (c). (d,e) The sample is then surrounded by grease, while the two platforms are pressed together by screws.

It was found that electrical resistivity of YHg_3_ and LuHg_3_ has metallic character (residual resistivity ratio, RRR = ∼400). This is supported by the band structure calculations, which indicate nonzero density of states at the Fermi level. Superconducting transitions below *T*_c_ = 1 ± 0.1 K for YHg_3_ and *T*_c_ = 1.2 ± 0.1 K for LuHg_3_ were observed in specific heat and resistivity measurements. As expected, a modest value of the Sommerfeld coefficient γ = 5–6 mJ mol_F.U._^–1^ K^–2^ was deducted from experimental data.

## Materials and Methods

**Figure 4:**
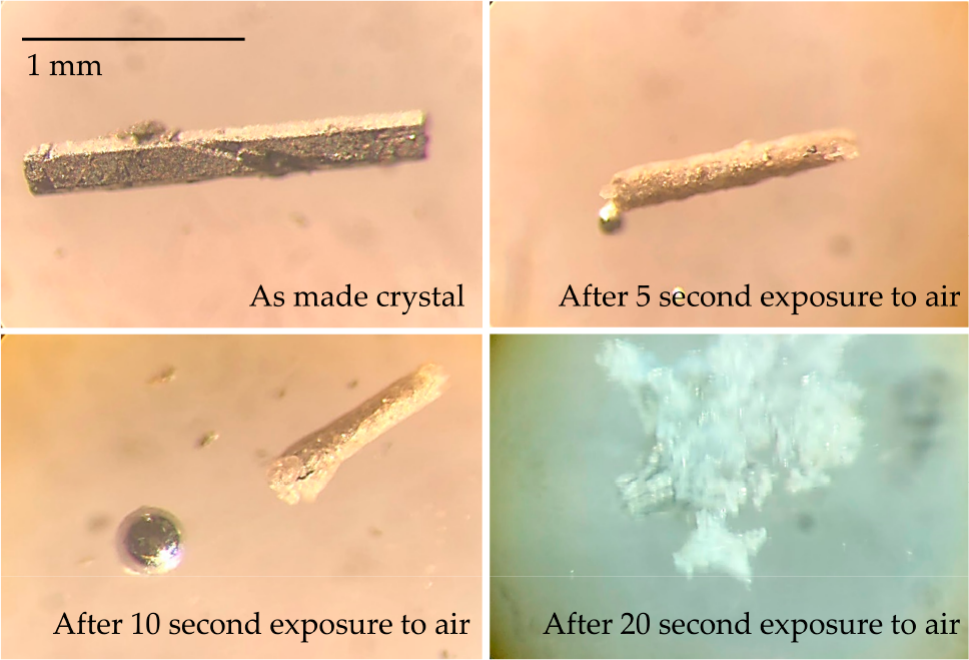
An approximate decomposition timeline of a LuHg_3_ single crystal upon exposure to air.

All sample preparation and handling was performed in a dedicated laboratory, equipped with an argon-filled glovebox system [MBraun, *p*(H_2_O/O_2_) < 0.1 ppm].^43^ Single crystals of YHg_3_ and LuHg_3_ were synthesized from Y/Lu (pieces, Ames Laboratory, >99.9%) and Hg (ChemPur, 99.999%) using the self-flux method. In order to separate crystals from the flux, a custom three-piece alumina crucible was used (see Figure 2b). In order to prevent mercury evaporation during synthesis, a thread was added to both the crucible and the strainer, compared with the earlier version.^44^ The Y/Lu chunks and Hg droplets, mixed in the 5:95 mass ratio (excess Hg), were sealed in Ta tubes under Ar atmosphere (Tantalum tubes were closed shut using an arc-melter, which was located inside a glovebox; Figure 2a). For other ratios of Y/Lu to Hg, single crystals could not be obtained. The volume of the Ta tubes was minimized so to exclude Hg loss (∼5 cm^3^ for a total sample mass of ∼10 g). The sealed Ta tubes were heated to 500 °C and then cooled to room temperature with a rate of 10 °C/hour (YHg_3_ and LuHg_3_) or 2 °C/hour (YHg_3_). Excess Hg flux was separated at room temperature via centrifugation. Some residual mercury could not be completely removed from the surface of the crystals. The resultant crystals had silver luster and needlelike morphology (see Figure 2c,d). Similar to the other mercury-based compounds,^15,16,36−42^ the YHg_3_ and LuHg_3_ phases exhibited extreme air- and moisture-sensitivity, thereby resulting in an immediate decomposition even after short exposure to air (Figure 4).

Powder X-ray diffraction was performed on a Huber G670 Image plate Guinier camera with a Ge-monochromator (Cu *K*_α1_, λ = 1.54056 Å). Phase identification was done using the WinXPow software.^45^ The lattice parameters were determined by a least-squares refinement using the peak positions extracted by profile fitting (WinCSD software^46^).

**Figure 5:**
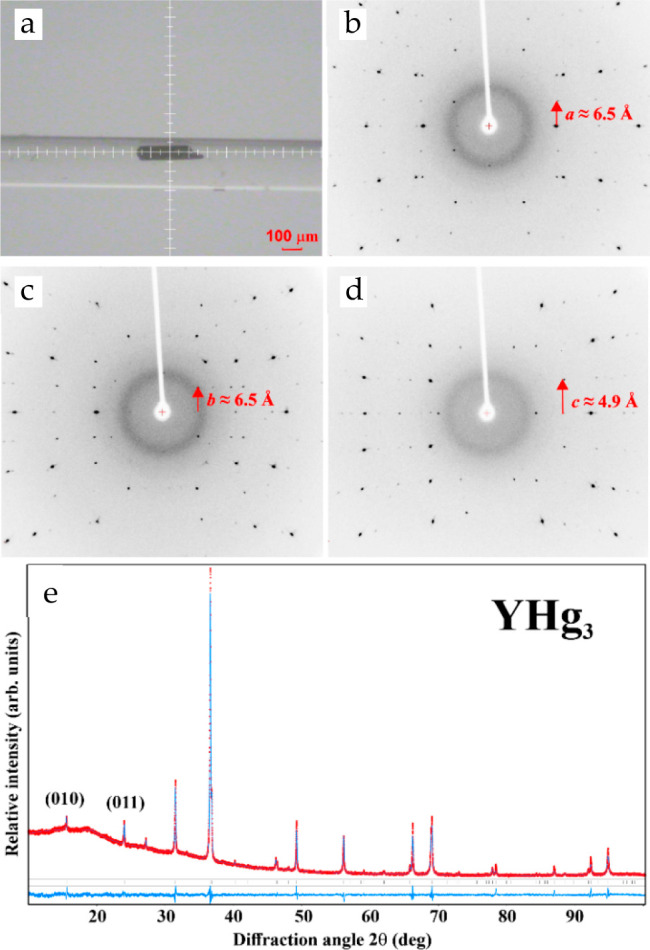
XRD characterization of YHg_3_: (a) single crystal in the capillary; a single-crystal partial oscillation XRD pattern around (b) [100], (c) [010], and (d) [001]. (e) An experimental powder XRD pattern (red symbols), together with the calculated profile (blue line) and the difference profile (blue line, bottom).

Differential thermal analysis (DTA) was performed on a Netzsch DTA 404 PC in the range from 30 to 700 °C in a sealed tantalum ampule in a steady Ar flow with a heating/cooling rate of 5 °C per minute. The resultant data are shown in Figure 5.

Small single crystals of YHg_3_ on the order of ∼50 μm were used for single-crystal diffraction experiments. Single-crystal diffraction data were collected using a Rigaku AFC7 diffractometer, equipped with a Saturn 724+ CCD detector and a Mo *K*_α_ radiation source (λ = 0.71073 Å). The WinCSD software^46^ was used for data analysis. The results of the crystallographic characterization are provided in Tables S1–S3.

Magnetic properties were studied using a Quantum Design (QD) Magnetic Property Measurement System for the temperature range from *T* = 1.8 K to *T* = 300 K and for applied magnetic fields up to *μ*_0_*H* = 7 T. Single crystals of YHg_3_ and LuHg_3_ were sealed inside quartz tubes, both to protect the sample from oxidation and to ensure sample orientation, with an example shown in Figure 2d. The specific heat data were collected on a QD Physical Property Measurement System (PPMS) in the temperature range from *T* = 0.4 K to *T* = 10 K for magnetic fields up to *μ*_0_*H* = 9 T. Extra grease was used to cover the sample in order to prevent decomposition. The corresponding contribution of the grease to the overall heat capacity was subtracted on the basis of the mass of the grease. The dc electrical resistivity measurements in a temperature range from *T* = 0.4 K to *T* = 300 K were carried out using a specially designed resistivity cell, shown in Figure 3. It consists of two sheets of poly(methyl methacrylate) (PMMA), which are held together by screws. Platinum wires are placed under the sample and then pressed against the sample surface. Grease is used to protect the samples from the environment. The other end of the wires is soldered onto a standard QD PPMS puck. An electric current was applied along the *c* axis of the single crystals, given the fixed position of the crystal with respect to voltage and current pairs. In mercury-based materials, a surface layer of mercury is frequently presenting an obstacle when measuring electrical resistivity. As summarized in Figure S1a, the intrinsic electrical resistivity of YHg_3_ and LuHg_3_ is different from that of pure Hg. In particular, in the case of pure mercury measurement, not only the superconductivity of mercury but also the structural transition of mercury from liquid to solid at *T* = ∼240 K can be observed. The *H*–*T* phase diagram of YHg_3_ and Hg is also significantly different, as summarized in Figure S1b.

Electronic structure calculations were performed by using the all-electron, full-potential local orbital (FPLO) method.^47^ All results were obtained within the local density approximation (LDA) to the density functional theory through the Perdew–Wang parametrization for the exchange–correlation effects.^48^ Application of the generalized gradient approximation did not reveal essential differences in the electronic structure below and in the vicinity of the Fermi level.

### Structure Description

The single crystals prepared as described above were characterized by X-ray diffraction techniques. Carefully ground crystals yielded a powder XRD pattern, which was fully interpreted with the hexagonal unit cell (*P*6_3_*/mmc* space group). The lattice parameters for YHg_3_ [*a* = 6.5443(5) Å and *c* = 4.8732(4) Å] and for LuHg_3_ [*a* = 6.465(1) Å and *c* = 4.848(2) Å] were in a good agreement with the values reported earlier (*a* = 6.541 Å and *c* = 4.87 Å,^10^*a* = 6.546 Å and *c* = 4.871 Å,^8^ respectively). The indexing of the powder diffraction data of YHg_3_ was confirmed by the Rietveld refinement, which resulted in low residuals (*R*_P_ = 0.007 and *wR*_P_ = 0.012). The (010) and (011) reflections indicate the ordering of Y and Hg in the basic atomic arrangement of the Mg type (space group *P*6_3_/*mmc*, *a* ≈ 3.27 Å, *c* ≈ 4.87 Å) and are clearly visible in the PXRD pattern (Figure 5).

**Figure 6:**
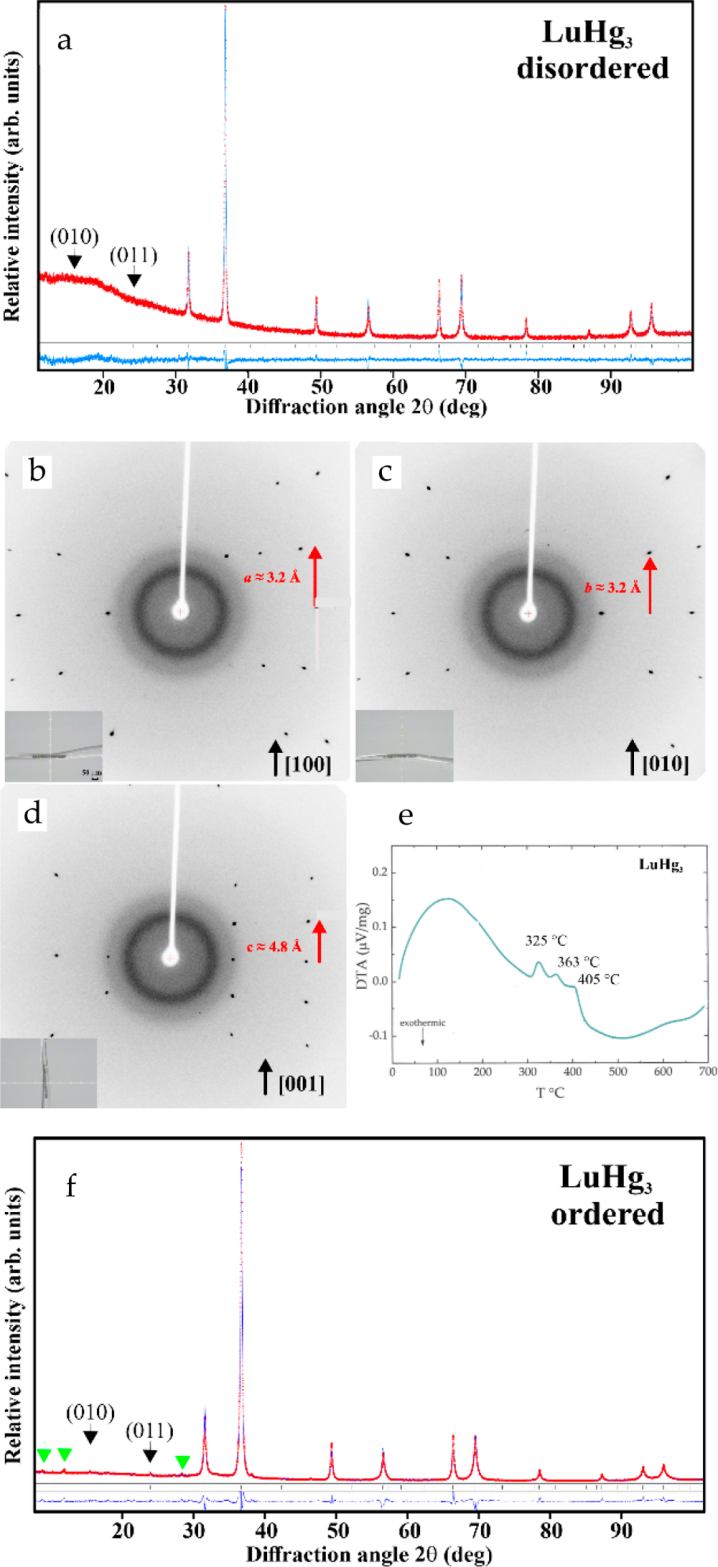
Order–disorder in LuHg_3_: (a) a powder XRD pattern of LuHg_3_ with a higher crystallization rate; note that the superstructure reflections are absent. A single-crystal partial oscillation XRD pattern around (b) [100], (b) [010], and (c) [001]. Insets show a single crystal in the capillary. (e) A DTA of the LuHg_3_ single crystals reveals several thermal effects. (f) A powder XRD pattern of the synthesis products with lower crystallization rate together with the calculated profile (blue line in the main panel) and the difference profile (blue line, bottom). Green arrows mark impurity phases (see main text for further details).

The PXRD pattern of pristine LuHg_3_ does not show the characteristic reflections [e.g., (010) and (011)], which indicates formation of the ordered Mg_3_Cd-type superstructure (Figure 6, top panel). This was also confirmed by the single crystal X-ray diffraction experiments (Figure 6b–d), which also did not reveal the superstructure reflections and indicated a disordered occupation of the positions in the Mg-type lattice.

The subsequent DTA study of the single crystals revealed a complex temperature-dependent behavior with several thermal effects (Figure 6e). Most of the phase transformations detected by DTA should be–at least partially–solid-state ones, e.g. formation of the polytype variants of the Mg_3_Cd type. Because the temperatures are relatively low, the reaction rates may, therefore, be reduced significantly. This should result in incompletely transformed structure patterns, thereby hindering the ordering of the crystal structure and requiring much smaller crystallization rates in comparison with those of YHg_3_.

After increasing the cooling time, the formation of the Mg_3_Cd-type superstructure was observed in the powder XRD pattern (Figure 6f). Nevertheless, additional weak diffraction reflections (Figure 6f, green arrows) were observed, which do not belong to any of the known binary phases. All attempts to index them using the structure data for the known superstructures of the Mg-type lattice (e.g., TiNi_3_ structure type, or apply automatic indexing) failed. This may indicate the presence of new phase(s) obtained as byproducts of the temperature-dependent reactions (cf. DTA data) or as result of sample decomposition during grinding. Additionally, this can signal the presence of some minor structural defects in the single crystals of LuHg_3_.

The single-crystal diffraction images for YHg_3_ are shown in Figure 5 a–d. Because of the high chemical activity and mechanical fragility of single crystals, they have to be used for experiments as grown, i.e., without any mechanical fragmentation (Figure 5a). This consequently requires a careful absorption correcting (linear absorption coefficient of 1450 cm^–1^). The details of the data collection and refinement are listed in Table S1, while the refined atomic coordinates and anisotropic displacement parameters are listed in Table S2. The obtained atomic displacement parameters show two striking features: strong anisotropy for the Y position (B33 ≫ B11) and larger displacement parameter for Hg with respect to the one of Y. Refinement of the crystal structure with the Y position shifted from the mirror plane on the [001] axis (z.ne.3/4) and the attempt to refine the occupancy of the Hg position are possible but both do not yield statistically relevant reduction of the residuals. Thus, the observed unusual features of the atomic displacement parameters may be caused mainly by the incompletely accounted influence of very high X-ray absorption (cf. huge value of the linear absorption coefficient). The structure refinement confirmed the atomic arrangement of the Mg_3_Cd structure type (ordered variant of the Mg structure type with *a* = 2*a*_Mg_ and *c* = *c*_Mg_; Y and Hg at the positions of Cd and Mg, respectively; *R*_F_ = 0.030, *wR*_F_ = 0.031).

The structural motif of YHg_3_ employs the hexagonal close-packed phase with the AB stacking sequence along the [001] direction. An ordered arrangement of Y and Hg atoms in the 1:3 ratio within each hexagonal layer (superstructure formation) requires doubling of the hexagonal lattice *a* in comparison with the “uni-color” packing (Mg structure type). This is evidenced by the appearance of superstructure reflections both in the single crystal and powder X-ray diffraction patterns (see Figures 5 and 6).

**Figure 7:**
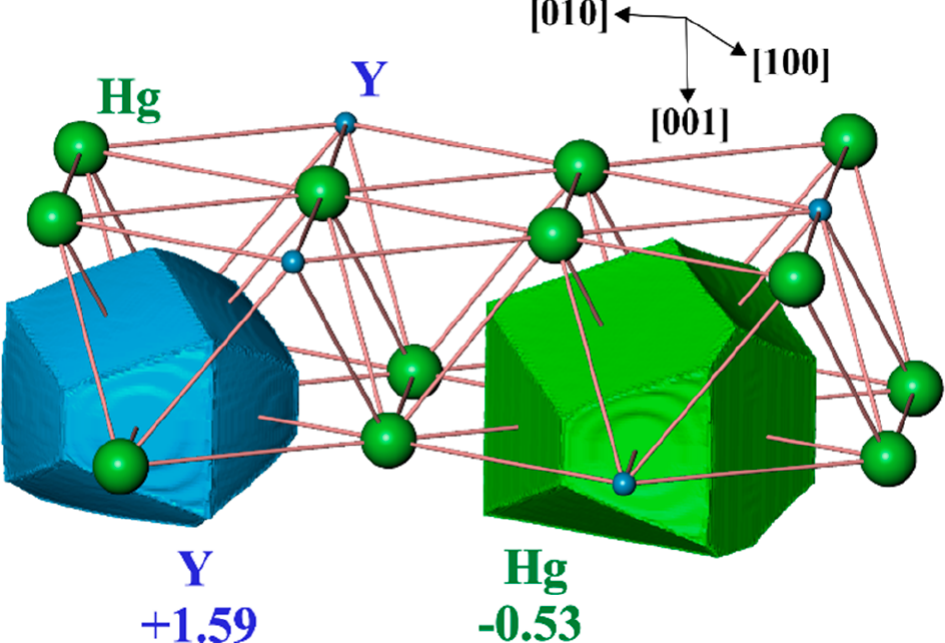
Shapes of the atomic QTAIM basins and their effective charges for YHg_3_. The shortest distances within and between the topologically closest packed layers are shown with pink lines.

Both species in the YHg_3_ structure have a coordination number of 12 and hexagonal analogues of cuboctahedra as their coordination polyhedra. Whereas Y atoms are surrounded exclusively by Hg atoms located at distances of 3.1085(6) and 3.2777(9) Å, the coordination polyhedron around Hg consists of 4 Y and 8 Hg species. The Hg–Hg distances of 3.0678(8) Å (4×), 3.219(1) Å (2×), and 3.335(1) Å (2×) are comparable with the values of 2.993 and 3.465 Å observed for octahedrally coordinated atoms in α-Hg (at *T* = 78 K).^49^ Calculation of the electron density (ED) and its analysis within the QTAIM approach reveal essential charge transfer from yttrium to mercury (Figure 7). The shapes of the atomic basins, obtained from the topological analysis of ED, show features characteristic of a charge transfer scenario. All faces of the QTAIM basin of the yttrium atom are convex toward the Hg ligands, which reflects in this way the difference in electronegativity (*EN*_Hg_ > *EN*_Y_). This behavior is similar to the recently investigated Mg_3-*x*_Ga_1+*x*_Ir^50^ and Mg_29-*x*_Pt_4+*y*_,^51^ where the more electropositive component Mg shows QTAIM basins with solely convex faces. Integration of the ED within the atomic QTAIM basins yields their electronic populations. Subtraction of the electron number for the neutral atom from the population yields the effective charge. The resultant charges of +1.59 for Y and −0.53 for Hg are in good agreement with the difference in electronegativities between these components. This also indicates that the possible disorder in the structure is most probably not caused by an exchange of Y and Hg at the respective crystallographic sites. This would be a clear energetic disadvantage as a result of an enhanced electrostatic contribution to the total energy of the system. Therefore, the origin of the disorder observed in the X-ray diffraction experiments is most probably coming from the stacking faults of the closest packed layers along the [001] direction.

## Superconducting Properties

**Figure 8:**
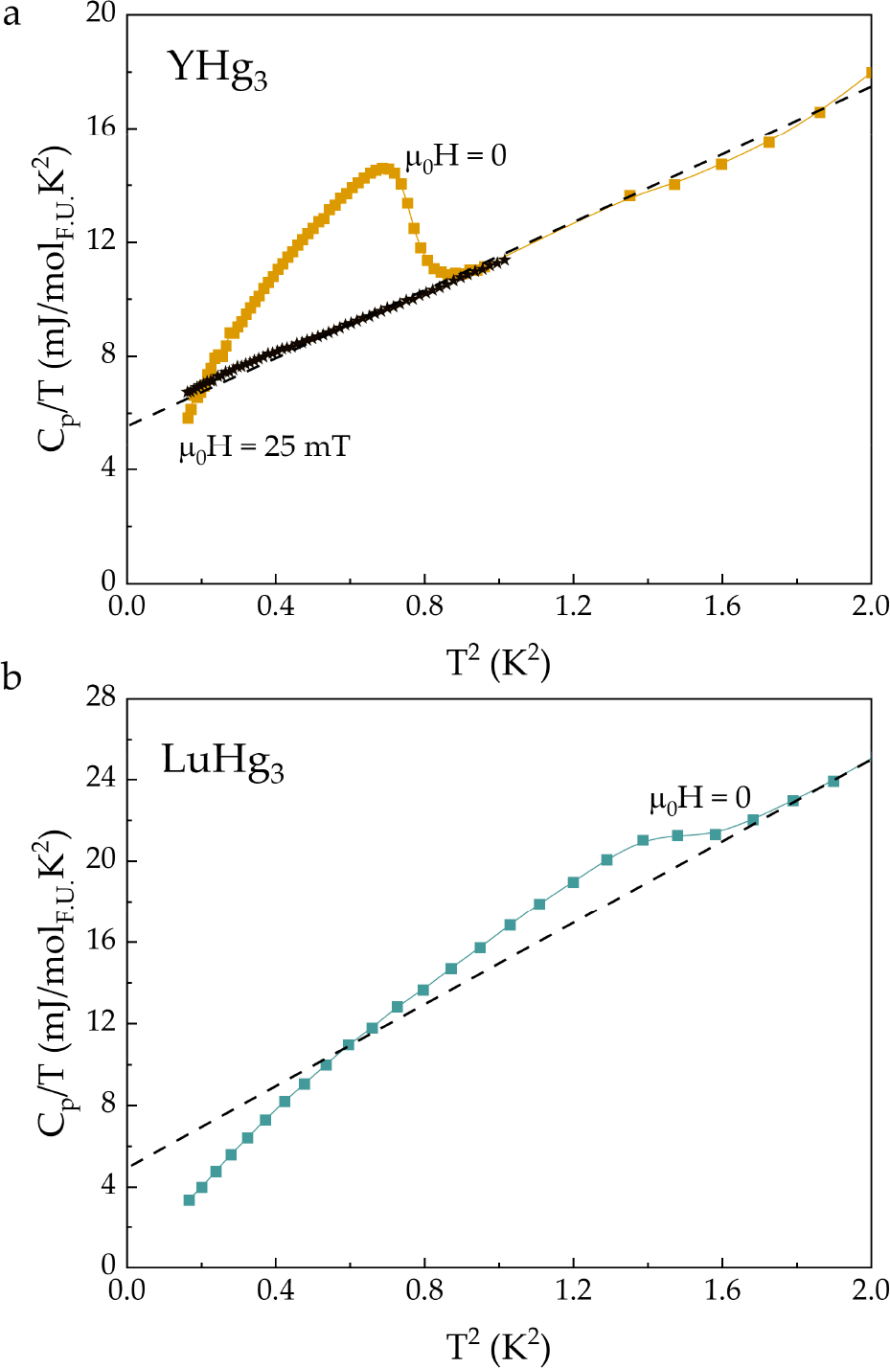
Specific heat of (a) YHg_3_ and (b) LuHg_3_, scaled by temperature, as a function of temperature squared. Anomalies corresponding to the superconducting transitions seen around *T*_c_ = 1 ± 0.1 K and *T*_c_ = 1.2 ± 0.1 K for YHg_3_ and LuHg_3_, respectively, are suppressed by the application of a magnetic field. An extrapolation of a linear fit above *T*_c_ yields a value of the Sommerfeld coefficient (γ_n_ = 5–6 mJ mol_F.U._^–1^ K^–2^), which is consisted with small effective electron mass of these materials.

The temperature-dependent magnetic susceptibility data of both YHg_3_ and LuHg_3_ indicate diamagnetic behavior down to the lowest measured temperature *T* = 1.8 K. However, a transition associated with an entrance into superconducting state is observed in the temperature-dependent specific heat data, shown in Figure 8a,b for YHg_3_ and LuHg_3_, respectively. The transition occurs at *T*_c_ = 1 ± 0.1 K for YHg_3_ and at *T*_c_ = 1.2 ± 0.1 K for LuHg_3_. Upon application of a magnetic field (Figure 8a), the transition is suppressed; however, given the small signal of the sample compared with that of the grease (which is necessary to prevent the sample from decomposition), the exact values of *T*_c_ for a given *H*_c_ value could not be established. Modest values of the Sommerfeld coefficient γ_n_ = 5–6 mJ mol_F.U._^–1^ K^–2^ for both YHg_3_ and LuHg_3_ were extracted from the linear fit of the *C*_p_/*T* vs *T*^2^ data in the normal state (dashed lines). Because of the difficulties of the background subtraction, it was not possible to estimate superconducting parameters for YHg_3_ and LuHg_3_, which would give more insights regarding the type of superconductivity in these compounds.

**Figure 9:**
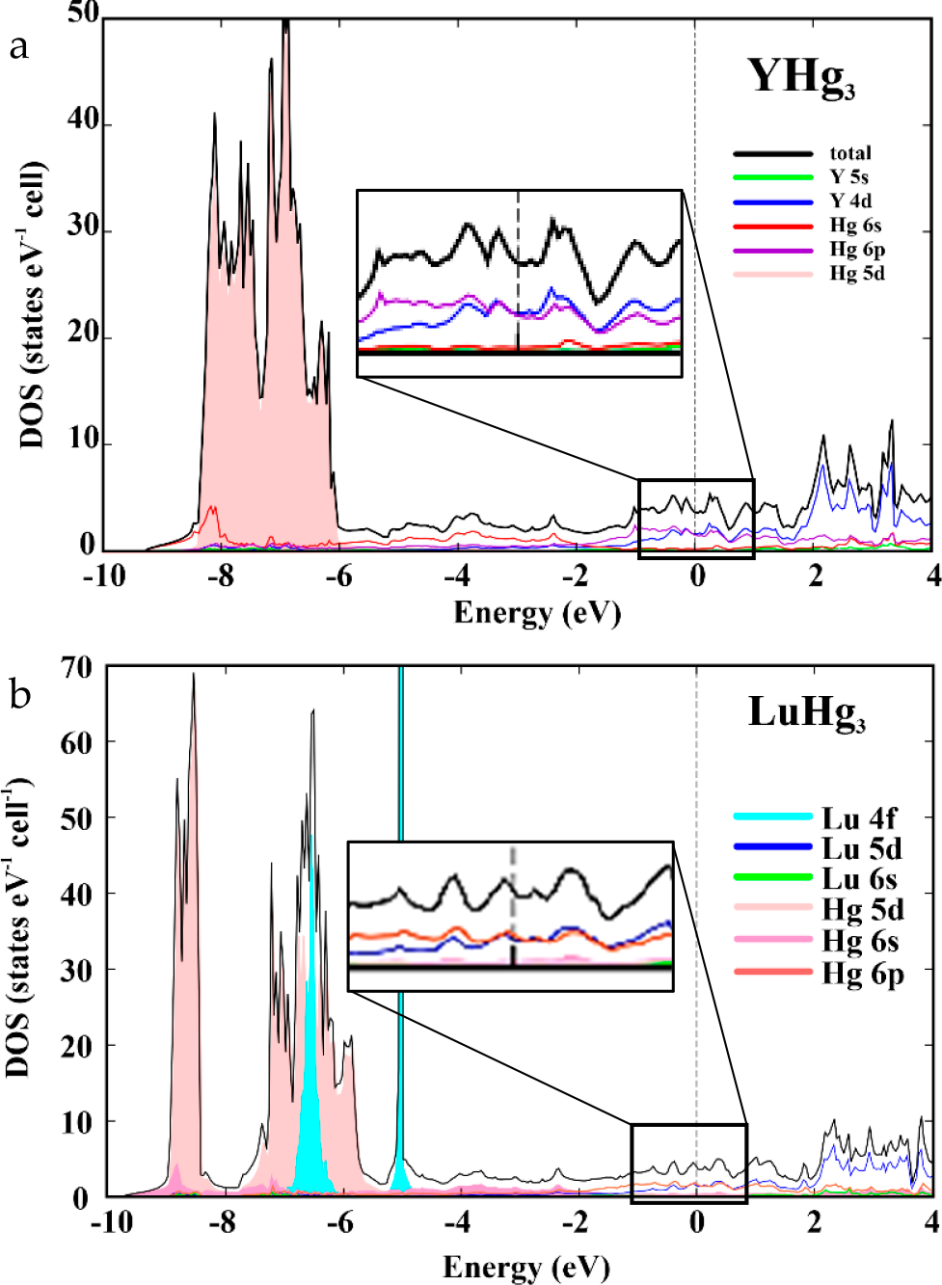
Calculated electronic density of states for (a) YHg_3_ and (b) LuHg_3_. The total density of states is shown in black, while the orbital- and element-specific contributions are shown in color. A finite density of states at the Fermi level is consistent with the metallic behavior of both compounds, which is observed in the resistivity data. Corresponding insets show a magnified view of the density of states close to the Fermi level.

Band structure calculations for YHg_3_ and LuHg_3_ are shown in Figure 9a,b, respectively. For both compounds, a nonzero density of states at the Fermi level is observed, from which the value of γ_theory_ = 5 mJ mol_F.U._^–1^ K^–2^ is estimated. This agrees well with the value of γ_n_ extracted from the specific heat data. It appears that at the Fermi level, the 4d orbitals of Y and 6p orbitals of Hg are dominant for YHg_3_ (see inset of Figure 9a). Similarly, the 5d orbitals of Lu and 6p orbitals of Hg are contributing the most to the overall density of states of LuHg_3_. The value of the density of states around the Fermi level remains constant for an extended energy range for both compounds, thereby suggesting that the ground states of YHg_3_ and LuHg_3_ are rather robust.

**Figure 10:**
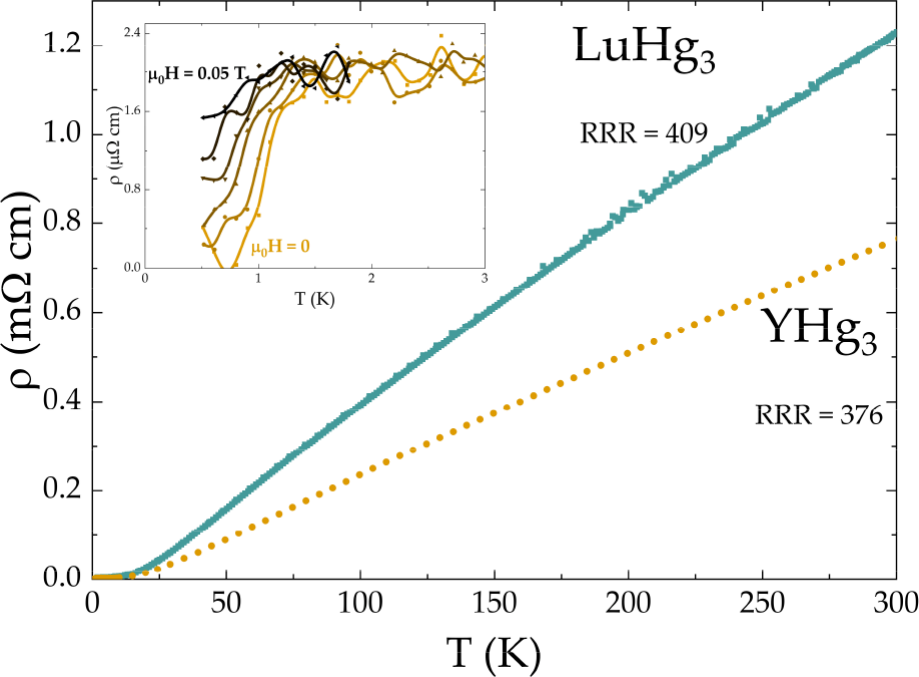
Temperature-dependent electrical resistivity of YHg_3_ (orange) and LuHg_3_ (blue) in μ_0_*H* = 0. High values (∼400) of the residual resistivity ratio (RRR) point toward a high sample quality for both compounds. The inset shows the low-temperature region of the resistivity data for YHg_3_. The position of the anomaly associated with the superconducting transition is gradually shifted by an application of an external magnetic field. The resistivity drop is no longer visible for μ_0_*H* > 0.05 T.

Metallic behavior is observed in the temperature-dependent electrical resistivity data of YHg_3_ and LuHg_3_, shown in Figure 10 and S1a. The metallicity of both systems is consistent with the nonzero density of states at the Fermi level observed in the band structure calculations. For both YHg_3_ (orange) and LuHg_3_ (blue), a high value of the residual resistivity ratio RRR ∼400 signals a good sample quality. The entrance into superconducting state is marked by a drop in the resistivity (Figure 10, inset), which is gradually moved to lower temperature upon application of a magnetic field. From the lower plateau of the transition, the corresponding *H*–*T* phase diagram is obtained, see Figure S1b. The resultant value of the critical field *μ*_0_*H*_c_(0) = 80 mT for YHg_3_ is estimated using a Ginzburg–Landau fit. Unfortunately, it was not possible to estimate the critical field of LuHg_3_ in a similar way, given its high air-sensitivity. However, it is rather likely that the value of the critical field in LuHg_3_ would be comparable with that of YHg_3_. Further in-depth analysis of YHg_3_ and LuHg_3_ using various methods, such as, for example, μSR^52,53^ would give more insights regarding their superconducting order parameter. Chemical substitution experiments could also be fruitful. Currently, studies of amalgams of rare-earth and actinides appear to be scarse.^11,24,38,39,54,55^ However, by using modern experimental tools, we are now able to conclusively unveil their peculiar chemical and physical properties.

## Conclusions

A detailed characterization of large single crystals of YHg_3_ and LuHg_3_ has been carried out. Because of the extreme air- and moisture sensitivity of these materials, it has only now been possible to definitively establish their crystallographic and physical properties. Both compounds crystallize in the Mg_3_Cd structure type [*P*6_3_*/mmc* space group, *a* = 6.5443(5) Å and *c* = 4.8732(4) Å for YHg_3_ and *a* = 6.465(1) Å and *c* = 4.848(2) Å for LuHg_3_). Low-temperature specific heat and resistivity measurements revealed the presence of superconductivity in both phases with *T*_c_ = 1 and 1.2 K, respectively.

## Data Availability

The data underlying this study are available in the published article and its Supporting Information.

## Supporting Information Available

Crystallographic data for YHg_3_ and LuHg_3_ (PDF)
