# Supplementary material for: Chemical and Physical Properties of YHg3 and LuHg3
Source: ACS Org Inorg Au. 2023 Feb 2;3(3):143–50. doi: 10.1021/acsorginorgau.2c00048 (PMC10251501; doi:10.1021/acsorginorgau.2c00048)
Supplement: Supplementary file 1 — gg2c00048_si_001.pdf [file gg2c00048_si_001.pdf]

Chemical and physical properties of YHg_3 and $LuHg_3$

Kristian Witthaut, Yurii Prots, Nazar Zaremba, Mitja Krnel, Andreas Leithe-Jasper, Yuri Grin, and Eteri Svanidze*

Max-Planck-Institut für Chemische Physik fester Stoffe, Nöthnitzer Str. 40, 01277 Dresden, Germany

[*] E-Mail: svanidze@cpfs.mpg.de

Table S1. Crystallographic data for YHg_3

Composition	YHg_3
Space group	$P6_3/mmc$
Pearson symbol	*hP*8
Formula units per unit cell, *Z*	2
Lattice parameters	
a / Å	6.554(2)
c / Å	4.8812(9)
V / Å^3	181.6(2))
Calc. density / g cm^{-1}	12.63
Crystal form*	rod-like
Crystal size / µm	25 × 45 × 110
Radiation, λ / Å	Mo$K\alpha$, 0.71073
Scan; step / degree; *N*(images)	φ, 1.0, 720
Maximal 2θ / degree	82.0
Range in *h*, *k*, *l*	$-10 \le h \le 9$
	$-10 \le k \le 9$
	$-2 \le l \le 8$
T(max)/*T*(min)	0.224
Absorption coeff. / mm^{-1}	145.0
N(*hkl*) measured	2496
N(*hkl*) unique	206
R_{int}	0.058
R_σ	0.0220
R_1 *(all)*	0.037
wR_2	0.070

$N(hkl)$ observed	187
Observation criteria	$F(hkl) \geq 4\sigma(F)$
Refined parameters	8
R_F	0.030
R_w	0.031
Residual peaks / e Å^{-3}	–0.95/1.60

Table S2. Atomic coordinates and displacement parameters (in Å^2) in the crystal structure of YHg_3

Atom	Site	*x/a*	*y/b*	*z/c*	U_{11}	U_{22}	U_{33}	U_{eq}
Y	2*d*	⅓	⅔	¼	0.0053(6)	U_{11}	0.0140(9)	0.0146(2)
Hg	6*h*	0.3275(1)	½*x*	¼	0.0118(4)	0.0106(3)	0.0130(3)	0.0152(3)

*$U_{12} = ½U_{11}$, $U_{13} = U_{23}$

Table S3. Crystallographic data for $LuHg_3$ (powder diffraction)

Composition	$LuHg_3$
Space group	$P6_3/mmc$
Pearson symbol	*hP*8
Formula units per unit cell, Z	2
Lattice parameters	
a / Å	6.4683(2)
c / Å	4.8517(2)
V / Å^3	175.794(7)
Calc. density / g cm^{-1}	14.67
Diffraction system	Huber Guinier G670
Radiation, λ / Å	Cu$K\alpha_1$, 1.54056
Maximal 2θ / degree	102.0
R_I	0.045
R_P	0.157
R_{wP}	0.178
Residual peaks / e Å^{-3}	–0.94/1.97

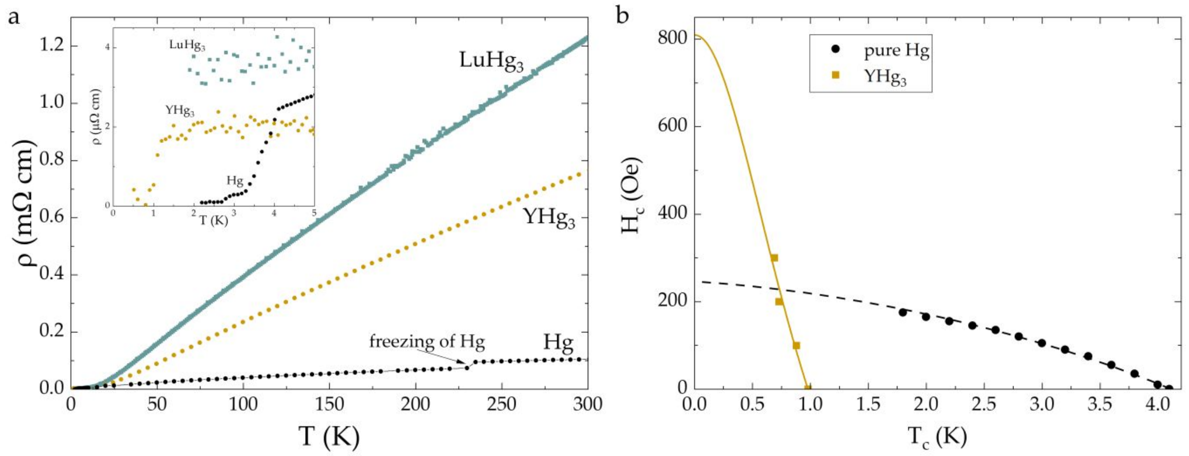

Figure S1: *(a) Temperature-dependent electrical resistivity of YHg_3 (orange), $LuHg_3$ (blue), and pure Hg (black) in $\mu_0H = 0$. The inset shows low-temperature region of the resistivity data. (b) The H-T phase diagram for YHg_3 (orange) and pure Hg (black).*
